# In Vivo Measurement of Cerebral Mitochondrial Metabolism Using Broadband Near Infrared Spectroscopy Following Neonatal Stroke

**DOI:** 10.1007/978-1-4939-3023-4_62

**Published:** 2015-06-22

**Authors:** Subhabrata Mitra, Gemma Bale, Judith Meek, Sean Mathieson, Cristina Uria, Giles Kendall, Nicola J. Robertson, Ilias Tachtsidis

**Affiliations:** 1 Institute for Women’s Health, University College London and Neonatal Unit, University College London Hospitals Trust, London, UK; 2 Biomedical Optics Research Laboratory, Department of Medical Physics and Biomedical Engineering, University College London, London, UK

**Keywords:** Broadband NIRS, Cytochrome c oxidase, Mitochondrial metabolism, Cerebral hemodynamics, Neonatal stroke

## Abstract

Neonatal stroke presents with features of encephalopathy and can result in significant morbidity and mortality. We investigated the cerebral metabolic and haemodynamic changes following neonatal stroke in a term infant at 24 h of life. Changes in oxidation state of cytochrome-c-oxidase (oxCCO) concentration were monitored along with changes in oxy- and deoxy- haemoglobin using a new broadband near-infrared spectroscopy (NIRS) system. Repeated transient changes in cerebral haemodynamics and metabolism were noted over a 3-h study period with decrease in oxyhaemoglobin (HbO_2_), deoxy haemoglobin (HHb) and oxCCO in both cerebral hemispheres without significant changes in systemic observations. A clear asymmetry was noted in the degree of change between the two cerebral hemispheres. Changes in cerebral oxygenation (measured as HbDiff = HbO_2_ − HHb) and cerebral metabolism (measured as oxCCO) were highly coupled on the injured side of the brain.

## Introduction

Perinatal stroke commonly presents with features of encephalopathy, seizures, or neurologic deficit during the early neonatal period. It can result in significant morbidity and severe long-term neurologic and cognitive deficits, including cerebral palsy, epilepsy and behavioural disorders. The incidence is high and has been estimated at 1 in 1600–5000 live births with estimated annual mortality rate of 3.49 per 100,000 live births [1]. Although seizures can be monitored with cerebral function monitor (CFM) or electroencephalography (EEG), the diagnosis of cerebral injury is typically confirmed on brain magnetic resonance imaging (MRI) once the infant becomes clinically stable [2, 3].

In contrast to adult stroke, the initial presentation of stroke in neonates can be subtle and non-specific. Neonates can present with lethargy, poor feeding, apnoea and hypotonia. This often delays the diagnosis and can influence the outcome. Any improvement in bedside non-invasive monitoring to aid early diagnosis and management would greatly benefit this group of infants.

Near-infrared spectroscopy (NIRS) is a non-invasive tool that has been widely used for continuous bedside monitoring of cerebral oxygenation and haemodynamic changes. NIRS can measure the concentration changes of oxygenated (Δ[HbO_2_]) and deoxygenated haemoglobin (Δ[HHb]) which in turn can be used to derive changes in total haemoglobin (Δ[HbT] = Δ[HbO_2_] + Δ[HHb]) and haemoglobin difference (Δ[HbDiff] = Δ[HbO_2_] − Δ[HHb]). HbT and HbDiff are indicative of cerebral blood volume and brain oxygenation, respectively. These measurements have been widely used to assess the haemodynamic changes in the cerebral tissue, but a clear assessment of cerebral metabolism during the same period is absolutely essential for a better understanding of the pathophysiology of cerebral injury and its management.

Cytochrome-c-oxidase (CCO) is the terminal electron acceptor in the mitochondrial electron transport chain (ETC). It plays a crucial role in mitochondrial oxidative metabolism and ATP synthesis and is responsible for more than 95 % of oxygen metabolism in the body [4]. CCO contains four redox centres, one of which—copper A (CuA)—has a broad absorption peak in the near-infrared (NIR) spectrum, which changes depending on its redox state [5]. As the total concentration of CCO is assumed constant, the changes in the NIRS-measured oxCCO concentration are indicative of the changes in CCO redox state in cerebral tissue, representing the status of cerebral mitochondrial oxidative metabolism.

Our group has recently demonstrated that brain mitochondrial oxidative metabolism measured by Δ[oxCCO] using broadband NIRS system during and after cerebral hypoxia-ischemia correlates well with simultaneous phosphorus magnetic resonance spectroscopy parameters of cerebral energetics in a preclinical model [6].

We have recently developed a new broadband NIRS system which is capable of absolute measurements of optical absorption and scattering to quantify Δ[oxCCO] as well as Δ[HbO_2_] and Δ[HHb] in neonatal brain [7]. In this study, we present the haemodynamic and metabolic changes following neonatal stroke. Our aim was to compare the haemodynamic and metabolic responses between the injured and non-injured side of the brain following neonatal stroke, using broadband NIRS measurement of changes in oxCCO.

## Methods

Ethical approval for the Baby Brain Study at University College London Hospitals NHS Foundation Trust (UCLH) was obtained from the North West Research Ethics Centre (REC reference: 13/LO/0106). We studied a term (40 weeks 6 days) newborn infant (birth weight 3370 g), admitted with clinical seizures. Seizures were first noted at 9 h of age and stopped at 17 h of age after treatment with multiple anticonvulsants (phenobarbitone, phenytoin, midazolam and paraldehyde). Seizures initially involved only the right upper and lower limbs. EEG recordings revealed repeated seizure episodes originating from the left hemisphere.

NIRS monitoring was commenced at 24 h of age. One NIRS channel was placed on either side of the forehead and data were collected at 1 Hz. Four detector optodes were placed horizontally against each source optode on either side with source-detector separations of 1.0, 1.5, 2.0 and 2.5 cm for multi-distance measurements. The longest optode source-detector distance of 2.5 cm was chosen to ensure a better depth penetration [8]. Differential path length (DPF) was chosen as 4.99 [9].

A program was created in LabVIEW 2011 (National Instruments, USA) to control the charge-coupled device (CCD), collect the raw data and calculate the corresponding concentrations. The changes in chromophore concentrations were calculated from the measured changes in broadband NIR light attenuation using the modified Beer-Lambert law as applied with the UCLn algorithm [10] across 136 wavelengths (770–906 nm). Systemic data from the Intellivue Monitors (Philips Healthcare, UK) were collected using ixTrend software (ixellence GmbH, Germany). Systemic and EEG data were synchronised with the NIRS data. Electroencephalography (EEG) data was collected using a Nicolet EEG monitor (Natus Medical, Incorporated, USA). Brain magnetic resonance imaging (MRI) and venography were performed on day 5 using a 3T Philips MRI scanner (Philips Healthcare, UK) on day 5. T1 and T2 weighted images with an apparent diffusion coefficient (ADC) map were obtained on MRI.

## Data Analysis

Initial data analysis was carried out in MATLAB R2013a (Mathworks, USA). NIRS data were visually checked and were processed with an automatic wavelet de-noising function, which reduces the high frequency noise but maintains the trend information. Systemic data were down-sampled and interpolated to the NIRS data timeframe (1 Hz). Artefacts from movement or changes in external lighting were removed using the method suggested by Scholkmann et al. [11]. This method also corrects shifts in the baseline due to artefact. All statistical analysis was performed using GraphPad Prism 6 (GraphPad Software, USA).

## Results

**Fig. 62.1:**
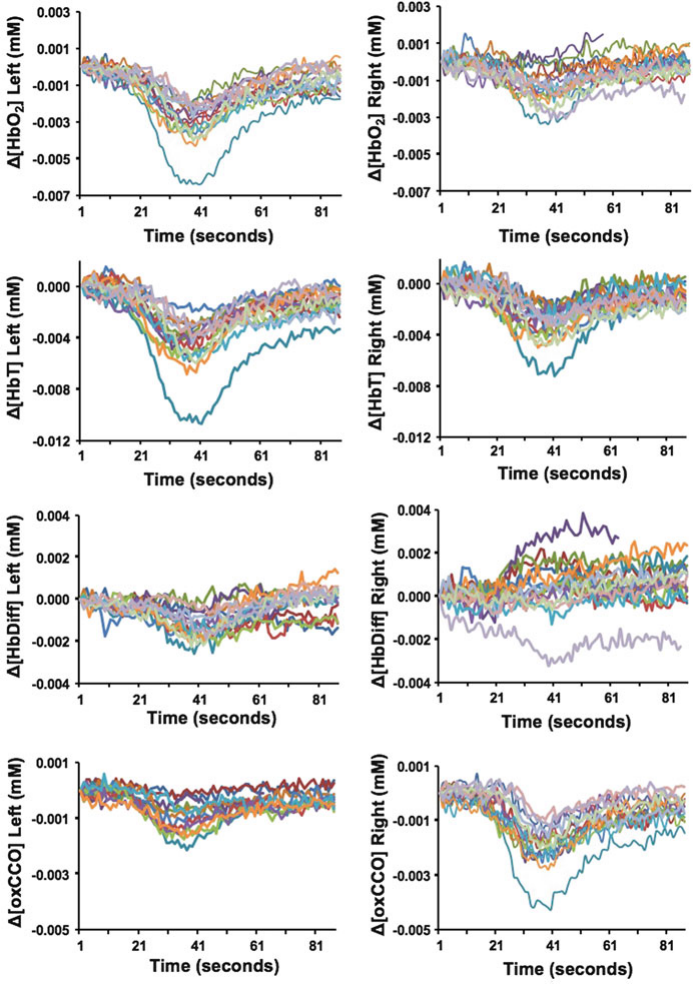
NIRSsignals from each side of the brain during all events (*each coloured line* represents a single event). Δ[HbO_2_], Δ[HbT], and Δ[HbDiff] reflect higher changes on the *left side*, but Δ[oxCCO] revealed minimal change on the injured left side compared to the right side

NIRS data were collected over a 3-h period without any clinical or electrographical seizure noted during this period. Synchronous and repeated transient changes in Δ[HbO_2_], Δ[HHb] and Δ[oxCCO] were noted on both sides (Fig. 62.1). Following an acute drop in these parameters, signals returned slowly towards baseline. These changes were noted over an average duration of 90 s. A change in Δ[HbT] of more than 2 μM was considered a significant event and 16 similar events were identified and analysed during the study.

**Fig. 62.2:**
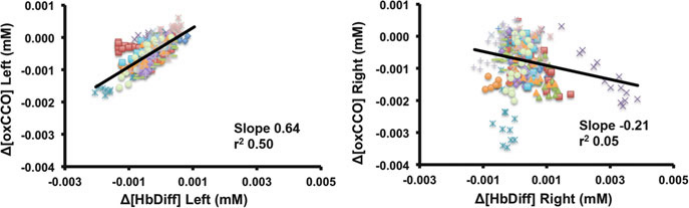
Linear regression analysis between Δ[oxCCO] with Δ[HbDiff] on both sides on day 1. Each coloured and different shaped point represents an event

A significant difference was noted between right and left sides in both cerebral metabolism, oxygenation and their relationship. Δ[HbO_2_], Δ[HbT] and Δ[HbDiff] were higher on the left (injured) side. However changes in [oxCCO] were more prominent on the right side during the events (Fig. 62.1). During the events, maximum concentration changes (fall) in Δ[HbO_2_], Δ[HbT], Δ[HbDiff] and Δ[oxCCO] were significantly different between the two sides (Table 62.1) but Δ[HHb] did not show any significant difference between the sides. Δ[oxCCO] responded differently to changes in Δ[HbDiff] between the left side (slope 0.64, r^2^ 0.5) and right side (slope −0.21, r^2^ 0.05) (Fig. 62.2).

**Table 62.1:** Differences in the maximum change between the left and right sides. Mean ± standard deviations of changes on both sides are presented with two-tailed p values

	Left	Right	p value
Δ[HbO_2_] (mmolar)	−0.0032 ± 0.0002	−0.0018 ± 0.0002	0.0002
Δ[HHb (mmolar)	−0.0020 ± 0.0001	−0.0020 ± 0.0002	0.9933
Δ[HbT] (mmolar)	−0.0049 ± 0.0004	−0.0036 ± 0.0003	0.0315
Δ[HbDiff] (mmolar)	−0.0016 ± 0.0001	−0.0008 ± 0.0001	0.0012
Δ[oxCCO] (mmolar)	−0.0011 ± 0.0001	−0.0021 ± 0.0001	0.0003

**Fig. 62.3:**
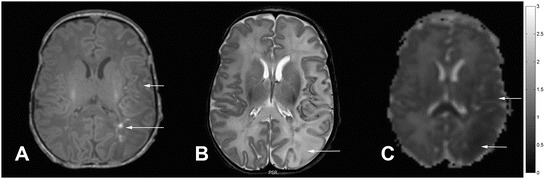
MRI scan taken at 3T on day 5. (**a**) T1 weighted axial image demonstrating generalised low signal intensity in the left parieto-occipital region with T1 shortening, (**b**) T2 weighted axial image demonstrating high signal intensity in the affected region with loss of cortical ribbon, (**c**) Apparent diffusion coefficient (ADC) map showing restricted diffusion in the affected area

MRI of brain on day 5 revealed low signal intensity on TI weighted images and high signal intensity on T2 weighted images in the left parieto-occipital region indicating a left sided neonatal stroke. Apparent diffusion coefficient (ADC) map demonstrated restricted diffusion in the same area on the left side (Fig. 62.3).

## Discussion

Spontaneous transient changes in NIRS parameters were recorded repeatedly from both cerebral hemispheres; a clear asymmetry was evident in these spontaneous haemodynamic and metabolic changes between the injured left side and the right side. The origin of these events is unclear. Similar events have been described previously following seizures using a different optical system [12]. We did not find any significant changes in systemic observations and electrical activity on EEG during the events in our study. Absolute band power in EEG was suppressed on the injured left side when compared to the right side during the study. It is possible that neuronal metabolic changes following seizures were driving the haemodynamic changes. Cerebral oxygenation (measured as HbDiff) and cerebral metabolism (measured as oxCCO) were tightly coupled on the injured side (left).

Following stroke, a persistent reduction in blood flow leads to a decrease in both substrate supply and oxygenation on the injured side [13]. These changes have opposite effects on Δ[oxCCO]. A decrease in substrate supply would lead to a change in redox state towards oxidation whereas a decrease in oxygenation will lead to a reduced redox state. These changes in redox state in opposite directions may explain why the Δ[oxCCO] response on the left side during these events was attenuated compared to the right side. The oxygenation and haemodynamic responses were however more exaggerated on the injured side. This restricted oxCCO change on the injured side of the brain is likely to reflect a persistent abnormal mitochondrial metabolism following unilateral seizures and reduced ATP turnover. An asymmetry in the cerebral energy state has been described with ^31^P MRS recorded from right and left cerebral hemispheres after seizures in a newborn baby [14]. This persisting abnormal cerebral metabolism may be due to the increased energy demand that occurs during persistent seizures; this is known to lead to unpredictable changes in the redox states of ETC metabolites [13].

In summary, we identified asymmetric cerebral oxidative and metabolic responses following neonatal seizures on day 1 using broadband NIRS measurement in a newborn infant. Although we were able to make an earlier predictive assessment, compared to the current standard clinical assessment tool (MRI) in this case study, a generalisation should be avoided at this point.

## References

[1] Lynch JK (2009). Epidemiology and classification of perinatal
stroke. Semin Fetal Neonatal Med.

[2] Lequin MH, Dudink J, Tong KA (2009). Magnetic resonance imaging in neonatal
stroke. Semin Fetal Neonatal Med.

[3] Govaert P (2009). Sonographic stroke templates. Semin Fetal Neonatal Med.

[4] Richter OM, Ludwig B (2003). Cytochrome c oxidase—structure, function, and
physiology of a redox-driven molecular machine. Rev Physiol Biochem Pharmacol.

[5] Jöbsis FF (1977). Noninvasive, infrared monitoring of cerebral and
myocardial oxygen sufficiency and circulatory parameters. Science.

[6] Bainbridge A, Tachtsidis I, Faulkner SD et al (2013) Brain mitochondrial oxidative metabolism during and after cerebral hypoxia-ischemia studied by simultaneous phosphorus magnetic-resonance and broadband near-infrared spectroscopy. Neuroimage pii: S1053-8119(13)00870-7 10.1016/j.neuroimage.2013.08.016 PMC4229502 23959202

[7] Bale G, Mitra S, Meek J et al (2014) A new broadband near-infrared spectroscopy system for in-vivo measurements of cerebral cytochrome-c-oxidase changes in neonatal brain Injury. Biomedical Optics, OSA Technical Digest, paper BS3A.39 10.1364/BOE.5.003450 PMC4206316 25360364

[8] Grant PE, Roche-Labarbe N, Surova A (2009). Increased cerebral blood volume and oxygen consumption
in neonatal brain injury. J Cereb Blood Flow Metab.

[9] Duncan A, Meek JH, Clemence M (1996). Measurement of cranial optical path length as a
function of age using phase resolved near infrared spectroscopy. Pediatr Res.

[10] Matcher S, Elwell C, Cooper C (1995). Performance comparison of several published tissue
near-infrared spectroscopy algorithms. Anal Biochem.

[11] Scholkmann F, Spichtig S, Muehlemann T (2010). How to detect and reduce movement artifacts in
near-infrared imaging using moving standard deviation and spline
interpolation. Physiol Meas.

[12] Cooper RJ, Hebden JC, O’Reilly H (2011). Transient haemodynamic events in neurologically
compromised infants: a simultaneous EEG and diffuse optical imaging
study. Neuroimage.

[13] Banaji M (2006). A generic model of electron transport in
mitochondria. J Theor Biol.

[14] Younkin DP, Delivoria-Papadopoulos M, Maris J (1986). Cerebral metabolic effects of neonatal seizures
measured with in vivo 31P NMR spectroscopy. Ann Neurol.

